# Gestational, perinatal, and postnatal factors that interfere with practice of exclusive breastfeeding by six months after birth

**DOI:** 10.1186/s13006-017-0132-y

**Published:** 2017-10-03

**Authors:** Mariana Moraes de Oliveira, José Simon Camelo

**Affiliations:** 1 Department of Pediatrics, Ribeirão Preto Medical School, University of São Paulo, Ribeirão Preto, São Paulo, Brazil; 2 Faculdade de Medicina de Ribeirao Preto, Departamento de Puericultura e Pediatria, Universidade de São Paulo, Avenida Bandeirantes, 3900, Monte Alegre, Ribeirao Preto, São Paulo, SP 14048-900 Brazil

**Keywords:** Breastfeeding, Exclusive breastfeeding, Weaning

## Abstract

**Background:**

Despite evidences indicating the superiority of breastfeeding and recent advances in the indicators of breastfeeding in Brazil, exclusive breastfeeding (EBF) during the first six months after birth continues to be an infrequent practice in the country. The objective of the present study was to determine which gestational, perinatal, and postnatal factors of the mother-baby dyad might be associated with the cessation of EBF by six months after birth.

**Methods:**

Data were collected at the rooming-in facility of the Reference Center of Women’s Health of Ribeirão Preto-Mater (CRSM-Mater) during the postpartum period (24 to 72 h after birth) from December 2012 to April 2013 and by telephone contact between the researcher and participating mothers by six months after birth.

Questionnaires were applied to collect data, such as the practice of EBF in the last 24 h in the sixth month after birth. The hierarchical theoretical model was proposed and data were analyzed statistically by log-binomial regression models using SAS 9.3.

**Results:**

The study involved 283 mother-baby dyads in which the mother evaluated did not present pregnancy-puerperal complications that could impede breastfeeding and confirmed the interest in breastfeeding her child. After the telephone contact in the exact sixth month after the birth of each participating baby, 84.8% of the participating mothers reported that they were no longer exclusively breastfeeding their babies. After statistical analysis, we found that there was a significant association between cessation of EBF and maternal report of previous experience with EBF for one month (0.91, 95% CI 0.81, 0.99) and six months (0.81; 95% CI 0.68, 0.94). These practices were associated with the maintenance of EBF and, even after adjustment for maternal socio-demographic variables, this association was maintained (0.85; 95% CI 0.73, 0.99). Thus, there is a greater chance of practicing and maintaining EBF by six months after birth when mother had previous experience with it.

**Conclusion:**

The identification of the risk variables associated with cessation of EBF by six months postpartum, such as previous experience with exclusive breastfeeding, may contribute to the effectiveness of EBF intervention and support measures during the first six months after birth.

**Electronic supplementary material:**

The online version of this article (10.1186/s13006-017-0132-y) contains supplementary material, which is available to authorized users.

## Background

Despite scientific evidence regarding the benefits and superiority of human milk (HM) as well as the harmful effects of infant formulas, the practice of exclusive breastfeeding (EBF) is still infrequent in Brazil. Even with the consolidation of numerous strategies to promote breastfeeding (BF) and the general public health recommendation that infants should be exclusively breastfed during the first six months of life in order to achieve good growth and development, the percentage of infants exclusively breastfed for the first six months of life is still low and worrisome [1–11].

A cross-sectional study conducted in the late 1990s in Brazil found alarming data on the estimated prevalence of EBF in the last 24 h by age of the child, showing that of 1259 children aged between 15 and 45 days of life, only 47.5% were In EBF [12].

In addition to this previous study, an important national survey entitled “National Survey of Demography and Health of Children and Women (PNDS) 2006” evaluated a sample of 4817 children considered to be representative of the Brazilian population and found that 42.9% were breastfed up to one hour after And that 48.3% of children aged 2–3 months were in EBF in the last 24 h [2].

The low EBF prevalence can be explained by sociocultural influences related to the industrialization process, such as the increasing commercialization of breast milk substitutes and the increase in female participation in the labor market. With the absence of women, the resources used to feed their children include bottles; milk from animal sources, such as cows; and industrialized infant formulas [10, 13–15]. The progressive substitution of breast milk as the sole source of nutrition, has had disastrous consequences for the health of babies since HM is a source of energy, nutrients, and bioactive factors, and is an environmental determinant of adequate intestinal colonization childhood, which favors the protection and defense of children against infections and diseases, as well as the physical, cognitive, and emotional development of the child [1, 4, 6, 16–21].

In addition, the low prevalence of BF in Brazil can be explained by the fact that BF is a biologically determined, yet culturally and socially conditioned, practice that makes the BF experience complex because it involves biological, psychological, and social factors related to both mother and their child [22–25]. Thus, considering that EBF can be influenced by factors related to the mother-baby dyad, the efficacy of intervention measures for BF support depends on the identification of risk factors for the cessation of EBF [10, 26].

Considering the above considerations, the objective of the present study was to determine which gestational, perinatal, and postnatal factors of the mother-baby dyad might be associated with the cessation of EBF by six months after birth. It is speculated that if such associations are confirmed, it will be possible to enrich prenatal and postnatal counseling to encourage EBF for a period of six months postpartum. Finally, we also sought to identify information that may help increase the prevalence of EBF in Brazil.

## Methods

### Study design

Data from this observational, analytical, and longitudinal study were collected in two stages. The baseline data collection involved the inpatient facility of the CRSM-Mater from December 2012 to April 2013. The follow-up data collection consisted of telephone contact between researchers and the participating mothers in the six months completed after the birth date (i.e., from May to September 2013).

In baseline data collection, clinical and sociodemographic information of the mother-baby dyad were collected through the application of a previously established questionnaire composed of open questions (see Additional file 1). When questioned about their previous experience with BF the mothers were provided a definition of EBF by the researcher conducting the interview. The interview of the participating mothers occurred in the joint housing of the CRSM - Mater during the postpartum period (24 to 72 h after birth) prior to discharge. In addition, the information contained in the questionnaire was confirmed using the records of the mother of the baby. Furthermore, information that the mothers were unable to answer was obtained through the clinical registry.

In follow-up data collection, a telephonic survey of the participating mothers was conducted by the researcher at six months after birth. This survey used a questionnaire to identify the type of feeding practiced at that time (see Additional file 2). This questionnaire was composed of two questions. In the first question, the mother was asked the following question regarding their practice of BF: Does your child ever breastfeed? In the case of a negative answer, it was considered that mother and baby no longer practiced BF, including EBF. In the case of a positive response, the investigator then informed the mother about the following definition of EBF, as indicated by the World Health Organization: EBF means that the baby receives only breast milk directly from the breast or pumped, or HM from another source, without any other liquids or solids, except for drops or syrups containing vitamins, oral rehydration salts, mineral supplements, or medications [27]. Next, the mother was questioned about their practice of EBF in the last 24 h: “Given the definition of EBF, do you practice EBF?” In the case of a negative answer, it was considered that mother and baby no longer practiced EBF. In subsequent statistical analyses, this answer was considered to indicate the cessation of EBF. In the case of a positive response, the mother was considered to be practicing EBF by six months after birth.

### Sample size

The sample size was calculated using the POWER procedure of the SAS 9.2 software and EBF prevalence rate of 11.5% related to 5–6 months infants born in Ribeirão Preto in 2011 [28]. Considering the birth of 1870 infants in the CRSM-Mater in the last six months from the study planning date, it was possible to assume prevalence rates and absolute errors (0.05) based on a 95% confidence level and thus estimate the sample size. Assuming that there would be a 10% loss of contact with the subjects interviewed six months after the date of birth, the sample size should be approximately 160 dyads. However, considering that the CRSM-Mater is intended to assist the female population that uses the National Health Unified System in Ribeirão Preto and some cities in the region, we decided to increase size of the sample since we assumed that we would lose contact with more than 10% of patients. Dyads were recruited and evaluated over a period of four months using the inclusion criteria for the first phase of data collection in the Maternal and Child Health Centers.

### Inclusion criteria

The babies selected for this study were only healthy, singleton term infants with a gestational age of 37 to 42 weeks who were born to healthy mothers with no gravidic-puerperal intercurrences that might prevent or hamper being breastfed [29, 30].

After the selection of the participating mothers and infants in the first stage of the study, the effective inclusion of these patients occurred after the researcher contacted the participating mother or legal guardian, in the case of adolescent mothers, provided information about the study procedures, and confirmed the interest of the mother in BF her child. Then, researchers obtained written informed consent.

### Statistical analysis

Data were analyzed statistically by fitting log-binomial regression models using SAS 9.3. Outcomes were defined as the cessation of EBF by six months after birth and the remaining gestational, perinatal, and postnatal factors of the mother-baby dyad were defined as independent variables. To test the associations between outcome and independent variables, a theoretical hierarchical model was proposed to stratify the relationship between variables and outcomes, as presented in Table 1. Therefore, the independent variables were arranged in hierarchical levels established according to the proximity of the outcome according to literature data [31]. Thus, four hierarchical levels were established: distal level, intermediate distal level, intermediate proximal level, and proximal level.

**Table 1:** Variables organized according to the temporal proximity to outcome

Distal level	Intermediate distal level	Proximal intermediate level	Proximal level	Outcome
Maternal sociodemographic variables	Prenatal care variables	Variables related to the period of delivery	Postnatal variables	Cessation of EBF by six months after birth
• Maternal age	• Planning of the last pregnancy	• Number of pregnancies	• Time of first baby suckling after birth	
• Maternal schooling	• Number of prenatal visits attended	• Period of birth	• Initial maternal difficulty with BF	
• Remunerated activity performed	• Maternal prenatal guidance about BF	• Classification of birth	• Maternal guidance about BF after birth	
• Maternal experience with EBF for at least one month				
• Maternal experience with EBF for at least six months				

In the statistical analysis, the variables of each level were adjusted in relation to the remaining variables of the same level and in relation to the variables present in the preceding hierarchical level. Thus, the variables of the distal intermediate level were adjusted in relation to the variables of the same level and in relation to the variables of the distal level. This process continued through the proximal level until the association of the variables with the outcome was established.

## Results

During the baseline data collection in the CRSM-Mater, 400 dyads were interviewed. After six months, during the follow-up data collection, telephone contact was possible and established with only 283 dyads. Thus, the loss of contact with the dyads was 29.25%.

The gestational, perinatal, and postnatal variables of the 283 dyads in the two research stages are presented in Table 2 and described below.

**Table 2:** Variables of the mother-baby dyads (*n* = 283) enrolled in this study

Variables of the mother-baby dyad	Total	%	Practicing EBF^a^	Cessation of EBF^b^
Maternal age at birth				
≤20 years	73	25.8	7	66
>20 years	210	74.2	36	174
Maternal schooling immediately after birth				
≤8 complete years of study	80	28.3	13	67
>8 complete years of study	203	71.7	30	173
Remunerated activity performed by the mother until birth				
No	167	59.0	30	137
Yes	116	41.0	13	103
Number of pregnancies				
Primigestae	121	42.8	12	109
Multigestae	162	57.2	31	131
Maternal experience with EBF for at least one month before birth				
No	152	53.7	17	135
Yes	131	46.3	26	105
Maternal experience with EBF for at least six months before birth				
No	216	76.3	24	192
Yes	67	23.7	19	48
Planning of the last pregnancy				
No	197	69.6	31	166
Yes	86	30.4	12	74
Number of prenatal visits attended				
< six	38	13.4	4	34
≥ six	245	86.6	39	206
Maternal prenatal guidance about BF				
No	147	51.9	23	124
Yes	136	48.1	20	116
Period of birth				
Daytime	143	50.5	15	128
Nighttime	140	49.5	28	112
Classification of birth				
Cesarean	111	39.2	16	95
Vaginal	172	60.8	27	145
Time of first baby suckling after birth				
During the first hour of life or before	109	38.5	17	92
After the first hour of life	174	61.5	26	148
Initial maternal difficulty with BF				
No	206	72.8	38	168
Yes	77	27.2	5	72
Maternal guidance about BF after birth				
No	12	4.2	2	10
Yes	271	95.8	41	230

^a^Mothers who responded positively to EBF practice in the last 24 h

^b^Mothers who responded negatively to EBF practice in the last 24 h

The participating mothers were 14 to 49 years of age, with 210 (74.2%) of the 283 mothers being 20 years of age or older; 203 (71.7%) reporting schooling of more than 8 years, and 167 (59.0%) reporting that they did not perform any type of remunerated activity until birth.

Clinical data collected immediately after childbirth revealed that 162 (57.2%) of the participating puerperal women were multigestae, 152 (53.7%) had no experience with EBF for at least one month, and 216 (76.3%) had no experience with EBF for at least six months.

In addition, 197 (69.6%) of the puerperal woman had not planned the last pregnancy, 245 (86.6%) participated in six or more prenatal visits, and 147 (51.9%) reported having received no guidance regarding BF during their prenatal care.

Perinatal clinical data showed that 143 (50.5%) neonates were born during the daytime period, 172 (60.8%) were born by vaginal birth, and 174 (61.5%) were breastfed for the first time only after the first hour of life.

A total of 206 (72.8%) did not report initial difficulties with BF and 271 (95.8%) reported having received BF instructions after birth.

After the telephone contact in the exact sixth month after the birth date of each participating baby, 43 (15.2%) mothers reported practicing EBF, however 240 (84.8%) mothers reported no more exclusively breastfeeding.

After statistical analysis of the data, the unadjusted logistic regression analysis indicated that the factors “Multigestae,” “experience with EBF for at least one month,” “experience with EBF for at least six months,” and “birth during the nighttime period” were associated with cessation of EBF by six months after birth; however, they behaved as protective factors for EBF, as presented in Table 3. The factor “mother reported difficulty with BF” was shown to be a risk factor for the cessation of EBF (Table 3).

**Table 3:** Logistic regression analysis using the theoretical hierarchical model proposed to stratify the variables (Table 1)

VariablesVariables	Crude Relative Risk	Distal level	Intermediate distal level	Intermediate proximal level	Proximal level
	CRR (95% CI)	RR1*(95% CI)	RR2*(95% CI)	RR3^a^(95% CI)	RR4*(95% CI)
• Maternal age > 20 years	0.91 (0.83, 1.01)	0.94 (0.83, 1.06)	–	–	–
• Maternal schooling >8 complete years	1.02 (0.91, 1.14)	0.98 (0.88, 1.10)	–	–	–
• Remunerated activity performed	1.08 (0.98, 1.19)	1.09 (0.97, 1.21)	–	–	–
• Multigestae	**0.9 (0.82, 0.98)**	–	–	0.97 (0.83, 1.15)	–
• Experience with EBF for at least one month	**0.91 (0.81, 0.99)**	1.01 (0.91, 1.12)	–	–	–
• Experience with EBF for at least six months	**0.81 (0.68, 0.94)**	**0.85 (0.73, 0.99)**	–	–	–
• Last pregnancy was planned	1.02 (0.92, 1.13)	–	1.01 (0.91, 1.13)	–	–
• ≥ six prenatal visits	0.94 (0.83, 1.06)	–	0.94 (0.76, 1.16)	–	–
• Mother reported that she did not receive prenatal guidance regarding BF	0.99 (0.9, 1.09)	–	0.99 (0.89, 1.11)	–	–
• Birth during the nighttime period	**0.89 (0.81, 0.98)**	–	–	0.95 (0.85, 1.06)	–
• Cesarean	1.02 (0.92, 1.12)	–	–	1.00 (0.89, 1.12)	–
• First suckling occurred after the first hour of life	1.01 (0.91, 1.12)	–	–	–	0.99 (0.84, 1.16)
• Mother reported difficulty with BF	**1.15 (1.05, 1.25)**	–	–	–	1.04 (0.91, 1.20)
• Mother received guidance about BF	1.02 (0.79, 1.32)	–	–	–	1.01 (0.76, 1.32)

e Relative Risk

RR* Adjusted Relative Risk

*95% CI* 95% Confidence Interval

Statistical analysis using the proposed theoretical hierarchical model (Table 3) revealed that only one variable was found to be associated with the outcome after adjustment according to the remaining variables. The results indicated that previous experience with EBF for at least six months before the birth reported by the mothers represented a protective factor EBF (i.e., the lack of previous maternal experience with EBF for at least six months is a risk factor for the cessation of EBF by six months after birth).

## Discussion

The present study was conducted in the city of Ribeirão Preto (SP) from 2012 to 2013 to evaluate 283 mother-baby dyads. In doing so, we detected a 15.2% prevalence of EBF among six-month-old babies (Table 2). A cross-sectional study conducted in Ribeirão Preto (SP) two years earlier revealed an EBF prevalence of 11.5% among 916 infants aged five to six months [28]. Despite detecting a positive difference in EBF rate, the prevalence of EBF in the city of Ribeirão Preto (SP), as well as in Brazil more generally, is still low and worrisome [2, 12, 32].

Several authors support and justify the reasons why it would be important to establish and maintain BF, particularly EBF, until the sixth month after birth. However, in view of the low BF rates in Brazil as a whole, these reasons are not sufficient to protect and promote BF practice [20, 33–36].

Several studies have emphasized their concern regarding the early interruption of the practice of BF in Brazil, particularly EBF, and point out possible associated factors, such as early motherhood, maternal educational level, mothers’ socioeconomic situation, maternal work outside home, parity, and lack of maternal knowledge about BF [28, 37–44]. In addition, the scientific literature reports that factors, such as number of pregnancies, pregnancy-planning, type of birth, and birth period, are also associated with BF establishment and maintenance before birth [26, 39, 45–47].

In the present study, unadjusted analysis revealed that a larger number of previous pregnancies were protective of the maintenance of EBF in the sixth month, (Table 3). Previous studies have reported that multiparous mothers and multigestae are more likely to perform BF than are primiparous mothers. They further demonstrated that primiparity is associated with an early interruption of BF [39, 48–52]. A study that conducted logistic regression analysis in order to test the association of risk factors with weaning reported that primiparous mothers had a greater risk of not providing EBF at 120 and 180 days after delivery. Regarding the period of 180 days after delivery, primiparity was shown to be associated with the interruption of EBF in terms of both gross (1.87; 95% CI 1.08, 3.24) and adjusted (2.20; 95% CI 1.14, 4.25) odds ratio [48].

One possible explanation for the association of parity with EBF is the role of previous maternal experience with BF. Studies have reported that women with previous BF experience have a greater chance of exclusively BF their babies in the first six months after birth [42]. A cohort study conducted on 1309 mother-baby dyads determined that the lack of previous BF experience was associated with the interruption of EBF as early as during the first month after birth (1.24; 95% CI 1.75, 1.43) [44].

In this study, we found that there was a significant association between cessation of EBF and maternal report of previous experience with EBF for one month (0.91, 95% CI 0.81, 0.99) and six months (0.81; 95% CI 0.68, 0.94). These practices were associated with the maintenance of EBF and, even after adjustment for maternal socio-demographic variables, this association was maintained (0.85; 95% CI 0.73, 0.99), as presented in Table 3. Thus, there is a greater chance of practicing and maintaining EBF until the sixth month after birth when mother had previous experience with it.

Despite the evidence obtained in the present study, a systematic review of epidemiological studies conducted in Brazil between 1998 and 2010, specifically 20 cross-sectional studies and 7 cohort studies, identified 36 factors associated with EBF. However, only two studies reported the association between prior maternal experiences with EBF by six months after birth [31]. In addition to this systematic review, a recent cohort study that was conducted in a city in the Northeast of Brazil in an effort to identify factors associated with the cessation of EBF did not find an association between the absence of prior BF experience and the cessation of EBF by six months after birth [53].

However, in addition to what has already been advocated, a recent study found that previous maternal experience with BF of the first child might determine the practice of BF the second child. However, the authors emphasized that mothers who had initial difficulties with BF their first child were less likely to initiate BF of the second child. Therefore, it is necessary to deal with the difficulties and quality of BF practice of their first child to ensure that the next child will be properly breastfed [54].

Therefore, we believe that it is important to emphasize that 152 and 216 mothers participating in our study reported that they did not have prior experience with EBF for one and six months, respectively. Because our sample comprised 121 primiparous women, who did not really have the possibility of previous BF experience, a higher number of mothers interviewed reported that they did not have such experience, which shows that some of the multigestae had no previous experience with EBF. Thus, we believe that previous experience with EBF influences BF practice and that the evaluation of the quality of this experience is necessary. Furthermore, it is important to evaluate the difficulties related to the process of BF previous children.

Regarding difficulties with BF, we did not question the reported previous difficulties with BF because its reporting was subjective and could have biased this study. However, we questioned the mother about initial difficulties with the current child’s BF but without detailing such a variable. After performing the unadjusted analysis of the data, responses to “initial maternal difficulty with BF” was associated with cessation of EBF by six months after birth (Table 3). Difficulties related to BF and inadequate practice of BF techniques are among the main reasons why mothers wean their children, thereby reinforcing the importance of specialized professional support to assist in the establishment and maintenance of BF [55, 56].

Finally, based on previous reports, the specialized support of health care professionals and the support of family members are fundamental to the practice of BF [57, 58]. Studies have indicated that the presence of the mother’s partner is associated with EBF practice during the first six months after childbirth and that better marital stability is related to a lower chance of early weaning [40, 41]. In addition, a systematic review showed improvement in EBF rates following individual and group interventions that supported BF [59]. Educational interventions, such as the Baby Friendly Initiative, that support, promote, and protect BF while simultaneously involving sectors, such as the home, community, and health system, have facilitated improvements in BF rates, particularly EBF, and favor the maintenance of this practice [60, 61].

We found an interesting association between the variable “period of birth” and the cessation of EBF. Possible speculation about night time birth as a protective factor for EBF could be the nature of the hospital environment, which is routinely calmer at night. This could more easily allow for skin-to-skin contact and BF during the first hour of life. In addition, visitors are only allowed to visit during the day, which may interfere with BF practice [62, 63].

### Limitations

The study presented some limitations such as the fact that information needed to justify the findings had not been collected, since there were dyads invited to participate in the study who refused the invitation after being informed of the procedures. In addition, the relatively small sample size and the restriction to a single data collection site can also be considered limiting factors of this study.

## Conclusions

The support of health professionals and society at large is essential to advocate the importance of EBF practice. However, being aware of the benefits and advantages of BF and opting for this practice are not enough to establish and maintain BF for an appropriate period.

It is important not to generalize the ability to breastfeed and to consider the variables involved in the BF process. Therefore, it is essential to promptly identify the risk variables associated with the early interruption of EBF, thereby contributing to the efficacy of intervention measures for the support of EBF during the first six months after birth. Our study allowed the identification of risk variables associated with the cessation of EBF by six months after birth, such as the previous experience with exclusive breastfeeding. We believe that the identification and consideration of variables associated with EBF cessation may contribute to the effectiveness of intervention and support measures related to breastfeeding.

When favorable interventions that promote BF are performed with consideration of different determinants of BF practice, EBF is more frequently practiced. This fact can provide economic and environmental advantages for children, women, and society, such as the reduction in health care costs [10].

## Additional files

Additional file 1: Description of data: Clinical and sociodemographic information of the mother-baby dyad were collected through the application of this previously established questionnaire composed of open questions. (DOCX 13 kb)

Additional file 2: Questionnaire used in the sixth month. Description of data: In follow-up data collection, this survey used this questionnaire composed of two questions to identify the type of feeding practiced at that time. (DOCX 17 kb)

## Abbreviations

BF: Breastfeeding
CRSM-Mater: Reference Center for Women’s Health in Ribeirão Preto-Mater
EBF: Exclusive breastfeeding
HM: Human milk
