# Supplementary material for: Gestational, perinatal, and postnatal factors that interfere with practice of exclusive breastfeeding by six months after birth
Source: Int Breastfeed J. 2017 Oct 3;12:42. doi: 10.1186/s13006-017-0132-y (PMC5627475; doi:10.1186/s13006-017-0132-y)
Supplement: Supplementary file 1 — Description of data: Clinical and sociodemographic information of the mother-baby dyad were collected through the application of this previously established questionnaire composed of open questions. (DOCX 13 kb) [file 13006_2017_132_MOESM1_ESM.docx]

| What is your current age? |
| --- |
| What is your current level of education? |
| Have you had any paid activity until the date of birth? |
| How many pregnancies did you have before the last one? |
| Do you have experience with EBF for at least one month? EBF means that the baby receives only breast milk directly from the breast or pumped, or HM from another source, without any other liquids or solids, except for drops or syrups containing vitamins, oral rehydration salts, mineral supplements, or medications [27]. |
| Do you have experience with EBF for at least six months? EBF means that the baby receives only breast milk directly from the breast or pumped, or HM from another source, without any other liquids or solids, except for drops or syrups containing vitamins, oral rehydration salts, mineral supplements or, medications [27]. |
| Did you plan the last pregnancy? |
| How many prenatal visits did you have? |
| While receiving your prenatal care, did you receive guidance about BF? |
| Was your child born during the day or night? |
| Was your child born via cesarean or vaginal birth? |
| How long after giving birth did you breastfeed your child? |
| Have you had any initial difficulties with breastfeeding? |
| Have you received advice about breastfeeding after giving birth? |

Additional file 1 - Clinical and sociodemographic information
