# Supplementary material for: Gestational, perinatal, and postnatal factors that interfere with practice of exclusive breastfeeding by six months after birth
Source: Int Breastfeed J. 2017 Oct 3;12:42. doi: 10.1186/s13006-017-0132-y (PMC5627475; doi:10.1186/s13006-017-0132-y)
Supplement: Supplementary file 2 — Questionnaire used in the sixth month. Description of data: In follow-up data collection, this survey used this questionnaire composed of two questions to identify the type of feeding practiced at that time. (DOCX 17 kb) [file 13006_2017_132_MOESM2_ESM.docx]

Additional file 2 - Questionnaire used in the sixth month.

| Does your child ever breastfeed? |
| --- |
| Yes. Definition of EBF, as indicated by the World Health Organization: EBF means that the baby receives only breast milk directly from the breast or pumped, or HM from another source, without any other liquids or solids, except for drops or syrups containing vitamins, oral rehydration salts, mineral supplements, or medications [27]. |
| No |
| Given the definition of EBF, do you practice EBF? |
| Yes |
| No |
